# Supplementary material for: Altered microRNA Transcriptome in Cultured Human Liver Cells upon Infection with Ebola Virus
Source: Int J Mol Sci. 2021 Apr 6;22(7):3792. doi: 10.3390/ijms22073792 (PMC8038836; doi:10.3390/ijms22073792)
Supplement: Supplementary file 1 [file ijms-22-03792-s001.zip › Supplementary_File/D_ KEGG_Analysis_Results/16-30nt_pathway_Reston-24h-Huh7_vs_Control-24h-Huh7_up.mature_mirna_targets/hsa_EnrichmentScore.pdf]

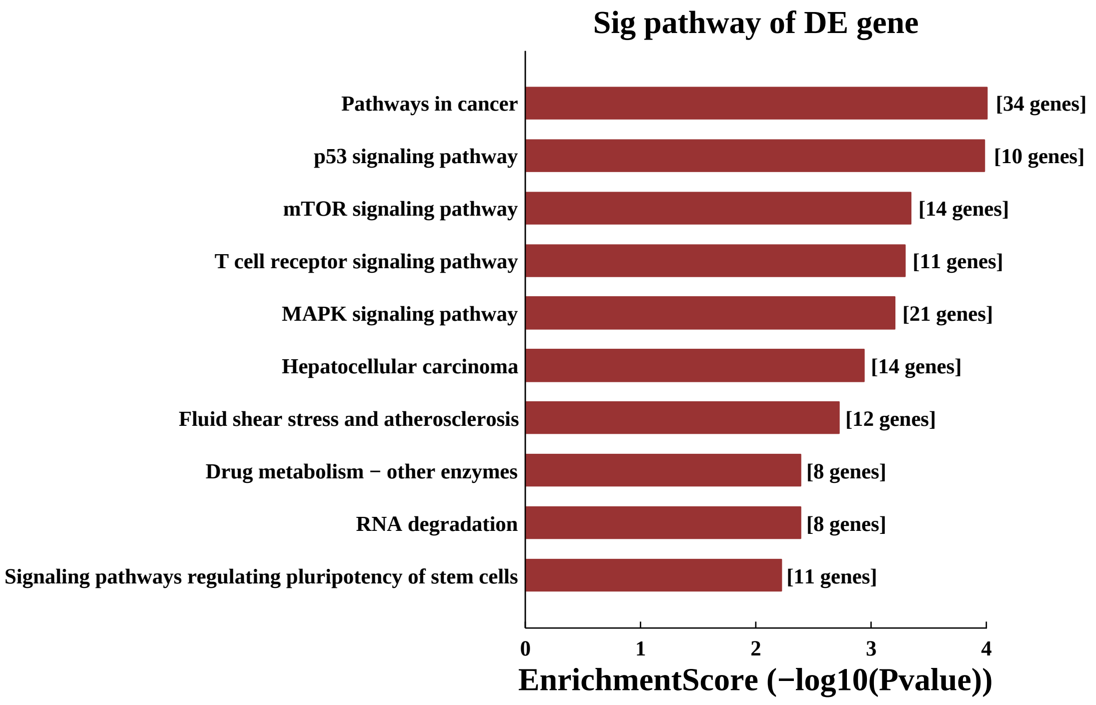

Sig pathway of DE gene
Pathways in cancer
p53 signaling pathway
mTOR signaling pathway
T cell receptor signaling pathway
MAPK signaling pathway
Hepatocellular carcinoma
Fluid shear stress and atherosclerosis
Drug metabolism − other enzymes
RNA degradation
Signaling pathways regulating pluripotency of stem cells
[34 genes]
[10 genes]
[14 genes]
[11 genes]
[21 genes]
[14 genes]
[12 genes]
[8 genes]
[8 genes]
[11 genes]
0
1
2
3
4
EnrichmentScore (−log10(Pvalue))
